# Chronic obstructive pulmonary disease as a risk factor in primary care: a Canadian retrospective cohort study

**DOI:** 10.1038/s41533-021-00249-5

**Published:** 2021-06-24

**Authors:** Andrew Cave, Anh Pham, Cliff Lindeman, Boglarka Soos, Tyler Williamson, Neil Drummond

**Affiliations:** 1 Department of Family Medicine, University of Alberta, Edmonton, AB Canada; 2 Faculty of Kinesiology, Sport, and Recreation, University of Alberta, Edmonton, AB Canada; 3 Department of Community Health Sciences, University of Calgary, Calgary, AB Canada

**Keywords:** Chronic obstructive pulmonary disease, Epidemiology

## Abstract

Chronic obstructive pulmonary disease (COPD) is a complex disease that is predicted to be the third most common cause of death by 2030. In Canada, the care and management of chronic conditions is largely provided by primary care providers. Although there is emerging research and initiatives that describe the prevalence of COPD in Canadian primary care settings, to our knowledge, there have been no efforts to use a large pan-Canadian database to analyze COPD as a risk factor for other common chronic conditions managed in primary care. We report the risk of developing comorbidities after the onset of COPD, that is, the extent to which COPD is a risk factor for developing common chronic conditions (heart failure, depression, anxiety, coronary artery disease, diabetes, anemia, hypertension, ischemic heart disease, underweight, and osteoporosis). After adjusting for age, sex, urban vs rural residence, and smoking status, the relative risks for patients with COPD at baseline were significantly higher for subsequent incidence of anemia, anxiety, diabetes, depression, heart failure, ischemic heart disease, lung cancer, osteoporosis, sleep apnea, underweight, and hypertension than patients without COPD. Using a cut-point of a 200% increase in relative risk as indicative of particular clinical relevance, COPD has a statistically and clinically significant association with developing lung cancer, becoming underweight, and developing heart failure.

## Introduction

Chronic obstructive pulmonary disease (COPD) is a common condition worldwide^1,2^. In Canada, it is more common in men than women and has a prevalence estimate in patients over the age of 40 years of approximately 6%^3,4^. It is associated with a variety of comorbidities^5^ that have an adverse effect on mortality and direct and indirect health care costs^6–8^. Some people believe that COPD and many of the associated conditions such as diabetes and coronary heart disease share a common inflammatory origin^9,10^. Several authors have described the prevalence and odds ratios (ORs) of conditions associated with COPD^3,11–13^. Less is known about the risk of developing comorbidities after the onset of COPD, that is, the extent to which COPD is a risk factor for developing those conditions^14,15^. Hence, we considered conditions that the literature indicated^5,14–17^ are associated with COPD and determined their rate of onset after diagnosis of the latter. Conditions selected for study had to have been reported as being associated with COPD and also available for analysis in the Canadian Primary Care Sentinel Surveillance Network (CPCSSN) dataset. For example, “frailty” is often reported as being associated with COPD; however, CPCSSN does not have a valid frailty definition and the condition was therefore not included in this analysis.

## Results

**Fig. 1:**
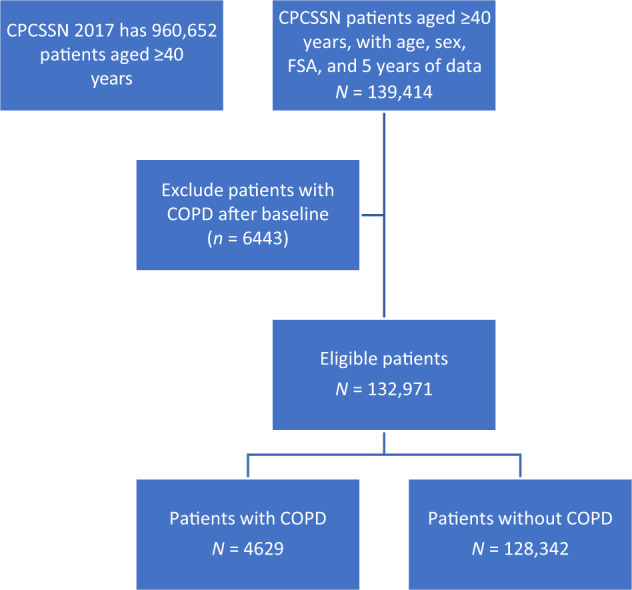
Derivation of Data for Analysis. Canadian Primary Care Sentinel Surveillance Network (CPCSSN), FSA (Forward Sortation Area), COPD (Chronic Obstructive Pulmonary Disease). Data available for analysis.

Of the 960,652 patients over the age of 40 years in the national CPCSSN^18^ as of December 31, 2017, 139,414 (14.5%) had complete age, sex, postal code (i.e., Forward Sortation Area (FSA)) and smoking data (free text) extracted from family physicians’ electronic medical records (EMR) after January 1, 1990, and at least 5 years of data (January 1, 2013–December 31, 2017). Of these, 4629 (3.3%) patients had a COPD diagnosis (by CPCSSN validated definition) prior to baseline (January 1, 2013) (Fig. 1).

### Patient demographic information

Patient demographic characteristics are presented in Table 1. Age, male sex, and smoking were significantly more common in patients with COPD at baseline compared to patients without COPD at baseline (*p* < 0.001). Urban residence is as common in COPD subjects as in non-COPD subjects (89.6 and 89.6%, respectively).

**Table 1:** Demographic characteristics.

	COPD at baseline (*n* = 4629)	Non-COPD at baseline (*n* = 128,342)	Test of significance
Age (±SD)	62.8 (11.2)	55.8 (10.9)	*t* test (*p* < 0.001)
Female (%)	2405 (52.0)	74,341 (57.9)	Chi^2^ (*p* < 0.001)
Urban (%)	4149 (89.6)	114,981 (89.6)	Chi^2^ (*p* = 0.008)
Smokers (%)	4175 (90.2)	89,943 (70.1)	Chi^2^ (*p* < 0.001)

Table 2 presents the adjusted and unadjusted relative risks (RRs) for incident diagnosis of anemia, anxiety, diabetes, depression, heart failure, hypertension, ischemic heart disease, lung cancer, osteoporosis, sleep apnea, and being underweight (body mass index <18.5) in COPD patients compared to non-COPD patients. The RRs were adjusted for patient age, sex, smoking status, and rurality. The adjusted RRs for patients with COPD at baseline were significantly higher for subsequent incidence of anemia (RR = 1.55; 95% confidence interval (CI) 1.44–1.67; *p* < 0.001), anxiety (RR = 1.34; 95% CI 1.28–1.41; *p* < 0.001), diabetes (RR = 1.15; 95% CI 1.09–1.22; *p* < 0.001), depression (RR = 1.68; 95% CI 1.59–1.76; *p* < 0.001), heart failure (RR = 2.64; 95% CI 2.40–2.90; *p* < 0.001), ischemic heart disease (RR = 1.44; 95% CI 1.35–1.55; *p* < 0.001), lung cancer (RR = 7.57; 95% CI 4.74–12.09; *p* < 0.001), osteoporosis (RR = 1.34; 95% CI 1.25–1.44; *p* < 0.001), sleep apnea (RR = 1.82; 95% CI 1.56–2.12; *p* < 0.001), being underweight (RR = 4.23; 95% CI 3.44–5.20; *p* < 0.001), and hypertension (RR = 1.10; 95% CI 1.06–1.16; *p* = 0.199).

**Table 2:** Relative risk for patients exposed to COPD compared to patients unexposed to COPD.

Comorbidity	Comorbidity incidence after baseline (%)	Unadjusted results				Adjusted results			
		RR	*p* value	Lower 95% CI	Upper 95% CI	ARR^a^	*p* value	Lower 95% CI	Upper 95% CI
Anemia	10,322 (7.8)	1.82	<0.001	1.69	1.96	1.55	<0.001	1.44	1.67
Anxiety	32,853 (25.9)	1.20	<0.001	0.97	1.08	1.34	<0.001	1.28	1.41
Diabetes	20,533 (16.1)	1.45	<0.001	1.39	1.53	1.15	<0.001	1.09	1.22
Depression	21,003 (16.3)	1.63	<0.001	1.55	1.71	1.68	<0.001	1.59	1.76
Heart failure	3527 (2.7)	4.77	<0.001	4.36	5.20	2.64^b^	<0.001	2.40	2.90
Hypertension	43,043 (35.5)	1.45	<0.001	1.40	1.49	1.10^b^	<0.001	1.06	1.16
Ischemic heart disease	12.237 (9.4)	2.19	<0.001	2.06	2.33	1.44^b^	<0.001	1.35	1.55
Lung cancer	90 (0.07)	11.89	<0.001	7.58	18.65	7.57	<0.001	4.74	12.09
Osteoporosis	13,421 (10.3)	1.81	<0.001	1.70	1.93	1.34^b^	<0.001	1.25	1.44
Sleep apnea	2734 (2.1)	1.81	<0.001	1.55	2.11	1.82	<0.001	1.56	2.12
Underweight^c^	834 (0.8)	4.12	<0.001	3.38	5.04	4.23	<0.001	3.44	5.20

^a^Relative risk for patients exposed to COPD compared to patients not exposed to COPD (adjusted for: age, sex, smoking status, and urban/rural postal code).

^b^Poisson distribution was applied and incidence rate ratios (IRRs) were produced instead of relative risk (RR).

^c^The median of all available BMI records on or after 01.01.2013 was used to compute a single BMI measure for each patient. Patients with a BMI under 18.5 were considered underweight; the analysis was conducted with the assumption that patients without an underweight BMI are not underweight.

## Discussion

In Canada, many patients with COPD are never seen in secondary care and only managed in primary care^11^. Based on a retrospective cohort study of primary care physician’s records, results showed that the elderly, males, and current or past smokers were more likely to develop COPD than those who are younger, female, or those who have never smoked. COPD onset had a statistically significant association with all outcomes of interest. However, using a cut-point of a 200% increase in RR as indicative of particular clinical relevance, COPD patients were statistically and clinically significantly associated with developing lung cancer, becoming underweight, and developing heart failure.

Our study confirmed a large number of conditions with known association with COPD. Chetty et al.^11^ assessed national records of over 52,000 patients in Scotland and found that 86% of COPD patients had at least one comorbidity compared to 49% of controls and over 22% had >5 comorbidities compared to only 5% of controls. He considered 31 physical conditions and 7 mental ones. Exploring the prevalence of individual comorbid conditions, he found an OR of 2.35 for heart failure in COPD but did not look at lung cancer or weight, nor did he consider incident risk, just association. As a validation, he found, as expected, no association between two unrelated neurological conditions, multiple sclerosis and Parkinson’s disease. We did not consider these because we do not have a validated CPCSSN definition for multiple sclerosis^19^, and for both conditions, the numbers in our sample were very small and analysis unjustified.

COPD was associated with increased RR for developing all of the conditions that we had drawn from the literature^5,14–17^. Soriano et al. reported a RR of 3.14 (95% CI 2.3–4.0) for osteoporosis (ours was 1.34 (95% CI 1.25–1.44); see Table 2). He also reported RR 1.75 (95% CI 1.2–2.5) for myocardial infarction and 1.67 (95% CI 1.4–2.0) for angina^14^. These may be compared to our use of the term “ischemic heart disease” (RR 1.44; 95% CI 1.35–1.55) and to the term “cardiovascular disease” used by Mannino et al. (OR 2.4; 95% CI 1.9–3.0)^15^. The latter also concluded that OR increased with the severity of COPD^15^.

The risks for lung cancer, becoming underweight, and heart failure, RR = 7.57, 4.23, and 2.64 (*p* < 0.001), were particularly evident in our findings. The high risk of developing lung cancer may be due to the common causative factor, smoking, though we adjusted for smoking as a possible confounder in the analysis, suggesting that a more nuanced interpretation may be required. Becoming underweight may itself be indicative of developing general frailty. Incident heart failure may similarly be indicative of multiple clinical system failure.

The American Academy of Family Physicians and the College of Family Physicians of Canada could not find strong enough evidence to support routine screening for lung cancer by low-dose computed tomography (LDCT)^20,21^, but, with a RR of 7.57, screening COPD patients for lung cancer might be considered to be indicated. The American Thoracic Society, the American Lung Association, and others recommend LDCT for high-risk patients who are aged 55–74 years and have a smoking history of 30 pack years based on the National Lung Screening Study^22^. This is reflected in the United States Prevention Strategies Task Force 2013 recommendation, which also includes current smokers (age 55–80 years) or those who smoked in the past 15 years^23^. Screening other groups is not recommended. The National Comprehensive Cancer Network 2014 suggest screening be extended to those who have a “personal history of lung disease: COPD, pulmonary fibrosis”^24^, and the Mayo clinic suggest “people with other risk factors for lung cancer” should be screened^25^. Our findings suggest that patients with COPD should be included in that group and have LDCT to detect early lung cancer while it is still treatable. While heart failure is always considered clinically as a differential diagnosis and comorbidity in COPD, the RR of osteoporosis we found would only weakly support the screening of COPD patients for osteoporosis but should raise clinician awareness of the increased risk of fractures.

## Methods

### Study design

This was a retrospective cohort study of patients with COPD receiving primary care in Canada between January 1, 2013 and December 31, 2017. Case definitions for COPD and comorbidities were derived from already validated case definitions^19,26^, from International Classification of Disease-9 codes recorded in billing, encounter diagnosis, or health condition/problem list tables in the EMRs, and from measurements such as body mass index. Table 3 lists the origin of each case definition. For reference, details of validated CPCSSN case definitions can be found at http://cpcssn.ca/wp-content/uploads/2014/07/CPCSSN_DiseaseDefinitionsFINAL_July16-2014.pdf^26^.

**Table 3:** Comorbidity case definitions.

Comorbidity	ICD-9 code(s)
Anemia	280–285
Anxiety	300
COPD	CPCSSN case definition
Diabetes	CPCSSN case definition
Depression	CPCSSN case definition
Heart failure	428
Hypertension	CPCSSN case definition
Ischemic heart disease	410–414
Lung cancer	162
Osteoporosis	733, 737.40–737.43, 737.7, 731.0
Sleep apnea	327.2, 780.51, 780.53, 780.57
Underweight	BMI between 5.0 and 18.5 (CPCSSN excludes BMI inputs <5.0 as an assumed error)

To ensure a robust study design with demographic information relevant to COPD and chronic disease comorbidities managed in primary care, strict requirements were enforced. To be included in the analysis, patients’ records must have included data for age, sex, FSA (to determine whether a patient lived in a rural or urban setting), smoking status (any instance of smoking behavior, whether light or heavy, current or past, deemed the patients as a lifetime “smoker” for the purposes of this analysis), and at least one primary care clinic encounter in 2013 and at least one in 2017. The baseline date (where the categorization of patients as COPD or non-COPD was made) was selected to ensure that there was a 5-year window when patients could present their first instance of a comorbidity. Without that restriction, there may have been an uneven distribution of time for patients to experience a comorbidity onset.

The study was granted approval by the University of Alberta, Research Ethics Office Pro:00079146.

### Data source

The CPCSSN database contains information from ten primary care research networks across the country; over 1300 participating primary care “sentinel” clinicians contribute data on >1,800,000 patients in 8 provinces and territories^18^. Primary care providers agree to have their EMR information extracted on a bi-annual basis for health services research, quality improvement, and disease surveillance purposes. To ensure patient and provider anonymity, patient and health care provider identifying data are removed at the time of data extraction and processing.

### Statistical analysis

We calculated the statistical differences in demographic data for patients with COPD at baseline compared to those without. Frequencies, means, and standard deviations are presented for continuous variables. Frequencies and percentages are presented for categorical variables.

Further, we calculated crude RRs for patients having COPD at baseline compared to patients without COPD, as well as RR adjusting for age, sex, smoking status, and urban/rural residence. The incidence of comorbidities was measured in those with COPD at baseline and in those without COPD at baseline during the 5-year follow-up period. When the binomial Generalized Linear Model was not concave, even after using *ml* and *irls* options to optimize the deviance^27^, Poisson distribution was applied to estimate RRs. Analyses were conducted with Stata 16^28^.

### Study limitations

This study is limited to the data available in individual primary care providers’ EMR. If a patient is new to a CPCSSN sentinel, it is possible that there is no data for that individual prior to their first encounter with that provider, thereby limiting the inclusion of data for the analysis. CPCSSN data does not include hospital-recorded information, limiting data to care received from their sentinel primary care provider alone.

CPCSSN has a validated algorithm for identifying conditions including COPD but not for all the comorbidities we examined (see Table 3). As a result, there may be variation in the misclassification rate due to physician diagnosis error and recording error among the comorbidities being considered. However, we anticipate no difference based on the COPD status of the patient. Further, some conditions may take many years to develop after COPD diagnosis and our cohort may not have continued long enough for them to present (e.g., CHD or anemia). In this latter case, the anemia of chronic disease may be offset by the anoxic drive toward polycythemia. However, the use of primary care data is also a strength of the study, in that it derives from a population which is representative of the population at large. Most COPD patients are managed in primary care only and so our results are highly relevant to this population.

This study assumed the first disease-specific record in the EMR data as identifying the incidence of a given condition and the order in which they developed. Due to differing latent periods, there is potential for error in disease indication.

Further, CPCSSN case definitions^26^ do not have 100% sensitivity^19^, and therefore there is a potential for the inclusion of false positive cases. Williamson et al. developed the case definitions with a utility threshold of 70%: case definitions that meet at least 70% sensitivity, specificity, positive predictive value, and negative predictive value are considered valid and sufficient for epidemiological studies^19^. We caution readers to potential misclassification of outcome^29^. Spirometry results were not available to confirm the diagnosis of COPD.

Lastly, only 14.5% of patients in the database were included in the cohort for analysis (Fig. 1). By insisting that all the cohort had complete data for the whole study period, we reduced the size of the cohort but increased its validity. We examined the cohort and found that it was comparable to its base population in terms of age, gender, and smoking.

### Reporting summary

Further information on research design is available in the Nature Research Reporting Summary linked to this article.

## Supplementary information

Reporting Summary

## Data Availability

The data that support the findings are available through a CPCSSN application process. Contact https://cpcssn.ca for more information.
